# Mortality due to cardiovascular diseases in the Americas by region, 2000-2009

**DOI:** 10.1590/1516-3180.2014.1322604

**Published:** 2014-04-01

**Authors:** Vilma Pinheiro Gawryszewski, Maria de Fatima Marinho de Souza

**Affiliations:** I MD, MSc, PhD. Advisor, Health Information and Analysis, Health Information and Analysis Unit, Pan-American Health Organization, Washington DC, United States; II MD, MSc, PhD. Advisor, Regional Health Observatory, Pan-American Health Organization

**Keywords:** Mortality, Cardiovascular disease, Trends [subheading], Inequality, Americas, Mortalidade, Doenças cardiovasculares, tendências, Desigualdades em saúde, Américas

## Abstract

**CONTEXT AND OBJECTIVE:**

Cardiovascular diseases are the leading cause of death worldwide. The aim here was to evaluate trends in mortality due to cardiovascular diseases in three different regions of the Americas.

**DESIGN AND SETTING:**

This was a time series study in which mortality data from three different regions in the Americas from 2000 to the latest year available were analyzed.

**METHODS:**

The source of data was the Mortality Information System of the Pan-American Health Organization (PAHO). Data from 27 countries were included. Joinpoint regression analysis was used to analyze trends.

**RESULTS:**

During the study period, the age-adjusted mortality rates for men were higher than those of females in all regions. North America (NA) showed lower rates than Latin America countries (LAC) and the Non-Latin Caribbean (NLC). Premature deaths (30-69 years old) accounted for 22.8% of all deaths in NA, 38.0% in LAC and 41.8% in NLC. The trend analysis also showed a significant decline in the three regions. NA accumulated the largest decline. The average annual percentage change (AAPC) and 95% confidence interval was -3.9% [-4.2; -3.7] in NA; -1.8% [-2.2; -1.5] in LAC; and -1.8% [-2.7; -0.9] in NLC.

**CONCLUSION:**

Different mortality rates and reductions were observed among the three regions.

## RESUMO

**CONTEXTO E OBJETIVO:**

As doenças cardiovasculares são as causas principais de morte em todo o mundo. O objetivo do estudo foi avaliar as tendências na mortalidade decorrente das doenças cardiovasculares em três diferentes regiões das Américas.

**TIPO DE ESTUDO E LOCAL:**

Este é um estudo de série temporal que analisa dados de mortalidade em três diferentes regiões das Américas, de 2000 até o último ano disponível.

**MÉTODOS:**

A fonte de dados foi a Sistema de Informação de Mortalidade da Organização Pan-Americana da Saúde (OPAS). Dados de 27 países foram incluídos. Utilizou-se *joinpoint regression* para analisar as tendências.

**RESULTADOS:**

Durante o período de estudo, as taxas ajustadas por idade padronizadas de mortalidade dos homens foram mais altas que a das mulheres em todas as regiões. As taxas da América do Norte (AN) foram inferiores que as dos países da América Latina (AL) e do Caribe Não Latino (CNL). As mortes prematuras (30-69 anos) foram 22,8% do total de mortes na AN, 38,0% na AL e 41,8% no CNL. A análise das tendências mostrou diminuição significativa nas três regiões. A AN acumulou a maior diminuição. A porcentagem média de mudança anual (AAPC) e respectivos intervalos de confiança de 95% foram -3,9% [-4,2; -3,7] na AN; na AL foi -1.8% [-2.2; -1.5]; e -1,8% [-2,7; -0,9] no CNL.

**CONCLUSÕES:**

Foram observadas diferentes taxas de mortalidade e diferentes reduções nas três regiões.

## INTRODUCTION

Cardiovascular diseases (CVDs) are the leading cause of death worldwide, accounting for 30% of the total number of annual deaths globally.^1^ They comprise major disorders of the heart and blood vessels, which include heart coronary disease and stroke. Nowadays, this group of diseases is not considered to be only a problem of developed countries, since estimates for 2010 showed that low and middle-income countries were more affected, since they accounted for 80% of these deaths.^1^

Globally, ageing and unhealthy behavioral changes like tobacco smoking, physical inactivity and unhealthy diets, have become important contributors to the increased prevalence of intermediate risk factors such as obesity, dyslipidemia, raised blood pressure and raised blood sugar. Age is an unavoidable risk factor, but avoidance of premature mortality should be a public health concern; the percentage of premature deaths ranges from 4% in high income countries to 42% in low-income countries.^2^

This group of diseases is important to public health not only because of their magnitude but also because of the possibility of intervention. Prevention strategies at population level to reduce circulatory system mortality rates can be classified as high-risk and community-based.^3^ The first of these relates to identifying individuals who are at high risk, through screening, and referring them for treatment. The second of these comprises implementation of programs at population level aimed at modifying one or more scientifically well established risk factors such as use of tobacco, physical inactivity, etc. As a result of implementation of such programs and policies, declines in mortality due to CVDs have been documented, mainly in developed countries but also in some Latin America countries.^4^ ^-^ ^8^

Socioeconomic indicators have been associated with differences in mortality, incidence and prevalence of cardiovascular risk factors,^1^ ^,^ ^7^ ^-^ ^8^ and considerable disparities among countries in the Americas regarding CVD death rates have also been found.^8^

## OBJECTIVE

The objective of this study was to evaluate trends in mortality due to CVD in three different regions of the Americas.

## METHODS

**Case definition and source of data**

Data were obtained from the Mortality Information System (updated in August 2012), which comprises mortality databases provided to the Pan-American Health Organization (PAHO) by the countries in the Americas. To ensure comparability among countries with different qualities of information, we used the data corrected for under-reporting and ill-defined deaths, in accordance with published methodology.9 This study included all the deaths for which the underlying cause of mortality was coded in Chapter IX, Diseases of the Circulatory System (I00-I99), of the International Statistical Classification of Diseases and Related Health Problems, Tenth Revision (ICD-10). Premature mortality was defined as deaths that occurred among people aged 30-69 years old. For this definition, the life expectancy in the countries of the region of the Americas10 and a publication from the World Health Organization (WHO)11 were taken into account.

The criterion for including countries in this study was that they needed to have a time series available for the study period. The countries were distributed geographically into three regions: 1) North America (2 countries): Canada and the United States; 2) Latin America (13 countries): Argentina, Brazil, Chile, Colombia, Costa Rica, Ecuador, El Salvador, Mexico, Nicaragua, Panama, Paraguay, Peru and Venezuela; 3) Non-Latin Caribbean countries (12 countries): Antigua & Barbuda, Aruba, Belize, Bermuda, Guyana, Montserrat, Saint Kitts & Nevis, Saint Vincent and the Grenadines, Suriname, Trinidad & Tobago, Turks & Caicos Islands and Virgin Islands (US). In addition to geographical location, these sub-regions have some economic, social and cultural similarity. The period of study included data from 2000 to the latest available year (LAY). For North America and Latin America, the LAY was 2009, and for the Non-Latin Caribbean it was 2008. 

Age-adjusted rates were calculated using estimates from the United Nations Population Division^12^ and the WHO Standard Population^13^ (direct standardization). This study analyzed anonymous secondary data on mortality and therefore no ethics approval was required. 

## Data analysis

This was a time-series study. We began with a descriptive analysis on 16,940,728 deaths due to CVD registered in the system, focusing on sex, age, sub-region of residence and year of occurrence. To explain the trend patterns, a subsequent analysis was performed using the Joinpoint Regression Program, version 4.0.1 (January 2013), from the National Cancer Institute (http://surveillance. cancer.gov/joinpoint/). The dependent variable was the age-adjusted rates and the independent variable was the year. This software identified points at which the slope of the linear trend changed significantly,5 by using the age-adjusted rates from 2000 to LAY to fit a regression line to the natural logarithm of the rates. It calculated the average annual percentage change (AAPC), which is a summary measurement of the trend over a fixed time period, taking into account the trend transitions.14 The AAPC was considered to be significant when the slope was different from "zero" at alpha = 0.05. The significance tests used a Monte Carlo permutation method.

## RESULTS

During the study period, in all the countries included in the study, females accounted for 51.0% and males for 49.0% of all CVD deaths. Table 1 presents some descriptive characteristics of this mortality in each region. The highest annual average of deaths was seen in North America, where almost one million deaths occurred annually (55.3% overall), followed by Latin America (around 800,000 deaths; 44.3% overall) and then Non-Latin Caribbean (around 7,500 deaths; 0.4% overall). The proportion of premature mortality varied widely regarding sex and region. Males showed a higher proportion of premature mortality in all sub-regions, compared with females. North America presented the lowest proportion of premature deaths (22.8% overall; 14.6% among females and 31.8% among males), in comparison with the other subregions. In Latin America, these percentages were: 38.0% overall; 31.3% among females and 44.3% among males. In the Non-Latin Caribbean countries, almost half of one percent of the deaths due to circulatory system diseases were considered to be premature (41.8% overall; 36.0% among females and 46.9% among males).

**Table 1.** Mortality due to cardiovascular diseases (ICD-10 I00-I99) according to sex and region. North America, Latin America and Non-Latin Caribbean, 2000-LA

	North America	Latin America	Non-Latin Caribbean
Deaths/year (average)			
Female	491,475	366,302	3,535
Male	445,775	383,784	3,952
Total	937,249	750,086	7,486
% proportion of deaths 30-69			
Female	14.6	31.3	36.0
Male	31.8	44.3	46.9
Total	22.8	38.0	41.8
Age-adjusted rates/100,000			
2000			
Female	159.9	193.4	243.3
Male	229.9	257.6	366.7
Total	192.3	222.9	296.4
LAY			
Female	109.3	163.1	216.4
Male	165.8	224.9	325.1
Total	135.5	191.4	264.1
% of change 2000-LAY			
Female	-31.6%	-15.6%	-11.0%
Male	-27.8%	-12.7%	-11.3%
Total	-29.5%	-14.1%	-10.9%

LAY = latest available year; North America (2009), Latin America (2009) and Non- Latin Caribbean (2008)

Although the average number of deaths was greater among females, the age-adjusted rates were not. In all regions, the rate among males was higher than the rate among female. Although the average number of deaths was greater in North America, the age-adjusted rates were not. Both in 2000 and LAY, the North American selected countries presented lower age-adjusted rates than the Latin American selected countries and Non-Latin Caribbean selected countries. Using the mortality rates for the latest year available, the risk of dying due to a CVD presented by a person who lived in the Non-Latin Caribbean region was 1.9 times the risk presented by a person who lived in the North American region. 

## Trend analysis

**Figure 1:**
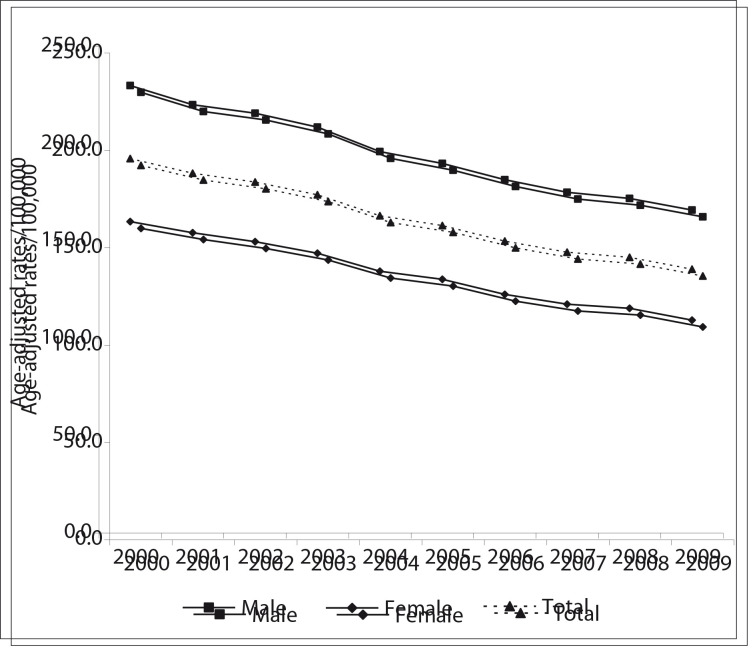
Trends in mortality due to cardiovascular diseases (ICD-10 I00-I99) (age-adjusted rates/100,000), in selected countries in North America (Canada and the USA), 2000-2009

**Figure 2:**
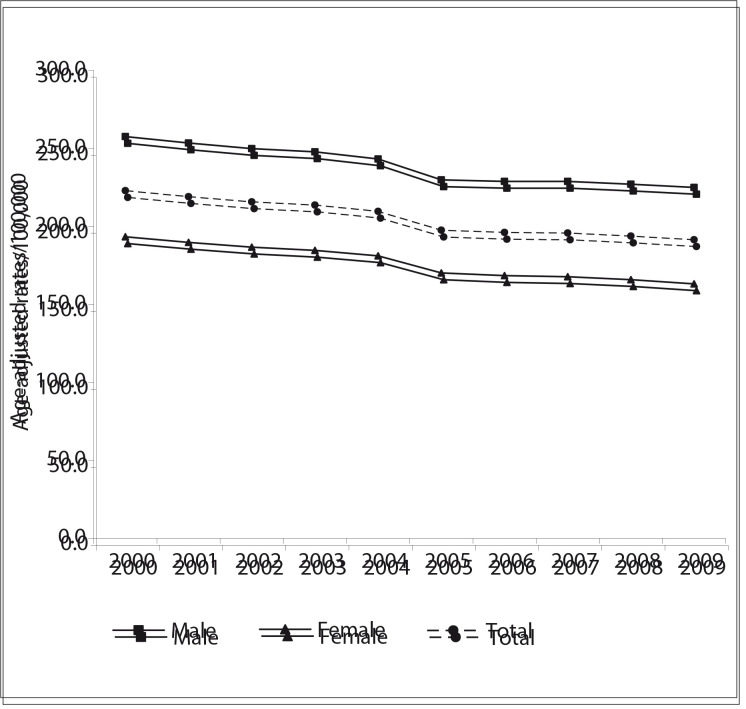
Trends in mortality due to cardiovascular diseases (ICD-10 I00-I99) (age-adjusted rates/100,000), in selected countries in Latin America, 2000-2009

**Figure 3:**
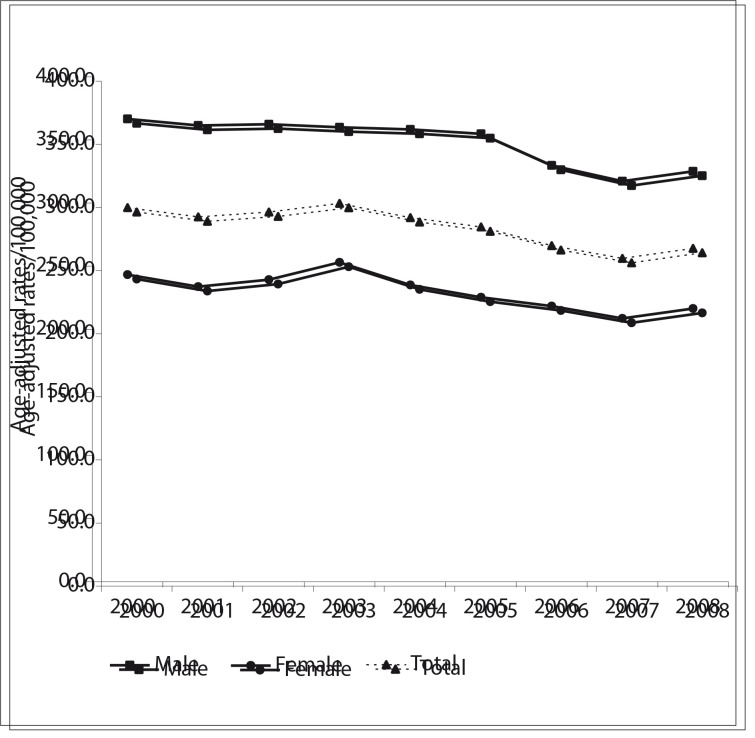
Trends in mortality due to cardiovascular diseases (ICD-10 I00-I99) (age-adjusted rates/100,000), in selected countries in Non- Latin Caribbean, 2000-2008

The results from the trend analysis (Table 1 and Figures 1, 2 and 3) showed that CVD mortality has declined in the three regions of the Americas. In North America, the overall ageadjusted rates per 100,000 dropped from 192.3 in 2000 to 135.5 in 2009, a decrease of 29.5%. The rates were higher among males but the decrease was greater among females. In Latin America, mortality has also been declining: the overall ageadjusted rates per 100,000 dropped from 229.9 in 2000 to 191.4 in 2009, a decrease of 14.1%. Compared with North America, the rates were higher and the decrease was smaller. In the Non- Latin Caribbean Region the adjusted rates per 100,000 dropped from 296.4 in 2000 to 264.1 in 2008, a decrease of 10.9%. The percentage decline was lower than in North America and Latin America. Although this region showed the smallest number of deaths, its age-adjusted rates were the highest. The trend curves for males and females followed the same pattern as shown by the total population curve (Figures 1, 2 and 3).

The results from the joinpoint analysis (Table 2) showed that CVD mortality in North America declined significantly from 2000 to 2009 and the corresponding AAPC was -3.9% (-4.3% among females and -3.7% among males). In Latin America, the AAPC was -1.8% over the entire period (-2.0% among females and -1.6% among males). In the Non-Latin Caribbean, the AAPC from 2004 to 2008 (the last 5 observations) was -1.8% (-1.8 % among females and -1.8% among males). 

**Table 2.** Joinpoint analysis for cardiovascular diseases (ICD- 10 I00-I99), according to sex and region. North America, Latin America and Non-Latin Caribbean, 2000-LA

	Years		AAPC [Confidence Interval]
	Lower Endpoint	Upper Endpoint	
Total			
North America	2000	2009	-3.9* [-4.2; -3.7]
Latin America	2000	2009	-1.8* [-2.2; -1.5]
Non-Latin Caribbean	2004	2008	-1.8* [-2.7; -0.9]
Female			
North America	2000	2009	-4.3* [-4.5; -4.0]
Latin America	2000	2009	-2.0* [-2.4; -1.6]
Non-Latin Caribbean	2004	2008	-1.8* [-3.0; -0.7]
Male			
North America	2000	2009	-3.7* [-3.9; -3.4]
Latin America	2000	2009	-1.6* [-2.0; -1.3]
Non-Latin Caribbean	2004	2008	-1.8* [-2.6; -1.0]

LAY = last available year

* The AAPC (average annual percentage change) is significantly different from "zero" at alpha = 0.05

## DISCUSSION

Our findings showed that mortality due to CVD has been decreasing in the three regions of the Americas, which is consistent with previous studies.^4^ ^-^ ^8^ However, there were marked differences among these regions. The North American countries, which have more favorable socioeconomic indicators,^9^ showed lower mortality rates, lower proportions of premature mortality and higher declines, in comparison with the selected countries in Latin American and the Non-Latin Caribbean.

The question that arises is what the reasons might be for the differences in the decline of CVD mortality. In Canada and the USA, mortality rates peaked in the mid-1960s and then experienced a long term decline.^4^ ^,^ ^5^ In the USA, approximately half of the decline in coronary disease mortality from 1980 to 2000 was attributable to clinical treatment (revascularization, initial treatment for acute myocardial infarction or angina and other treatments) and approximately half to changes in risk factors (reductions in total cholesterol, blood pressure, smoking and physical inactivity).^15^ For some Latin America countries for which published trend series studies are available, the decline took place recently, or there was no decline or even an increase.^4^ ^-^ ^5^ ^,^ ^8^ These countries have probably implemented community-based prevention programs more recently.

In addition, populations that live in countries with more disadvantaged incomes might face greater difficulties in obtaining access to treatment, compared with those who live in North American countries. Some countries in the Americas, such as Argentina, Brazil, Cuba and Canada, have public universal health coverage. Despite this, in Brazil, a study found that premature mortality due to circulatory system diseases was 2.6 times higher in poor areas in Porto Alegre than in rich areas.^16^ In São Paulo, the decline in the risk of death due to heart disease was faster for people living in the wealthiest areas and slower for people living in lower-resourced neighborhoods.^17^ In the United States, which has a different health system and the percentage health insurance coverage is around 83%, a study showed racial disparities in use of coronary artery bypass grafting and percutaneous transluminal coronary angioplasty among elderly women and men, which was probably due to access to care and financial barriers.^18^

Regarding risk factors, a lot of work has to be done in the region. In the USA, around 90% of the people consume sodium at levels above the recommended guidelines.^19^ The prevalence of an abnormally large abdominal waist circumference among people aged 20 years and over was around 70% in San Salvador, El Salvador (52% among males and 79% among females), and 64% in Belize (20% among the white population and 63% among the black and mixed population).^20^ In Brazil, the prevalence of medical diagnoses of hypertension among the adult population reached 23% in 2010 (26% among females and 21% among males).^21^ On the positive side, prevention activities have been implemented in many countries. Argentina, Barbados, Bolivia, Brazil, Chile, Cuba, Dominica, Mexico, Panama and Venezuela have implemented CVD and hypertension control programs.^22^ Tobacco policies have been implemented in 13 countries in Latin America. Mexico, Brazil, Chile, Costa Rica, Dominica, Paraguay and Peru have implemented initiatives to regulate the marketing of foods to children.^23^

It should be noted that the CVD burden goes beyond mortality. It has social and economic impacts such as increased costs of healthcare, disabilities and losses in productive years of life due to premature mortality. For example, in the United States, the estimated cost of medical care for CVDs in 2009 was around US$ 314 billion, and the indirect costs due to disability and deaths was around US$ 161 billion, which raised the total cost of these diseases to US$ 475 billion.^23^

The main limitations of the present study are the following: 1) although the rates were corrected for under-registration of deaths and ill-defined causes, it is possible that these problems might affect the rates in some countries, especially those with lower resources; and 2) some lower-resourced countries have made efforts to improve the quality of data, including decreasing the numbers of ill-defined deaths, which might lead to an increase in circulatory system disease rates. 

Since disease is a great contributor to poverty, the Americas should strive to bridge the gap in treatment and preventive control of CVD among its regions. Gold-standard prevention requires complementary clinical and community approaches and monitoring information regarding mortality, morbidity and prevalence of risk factors. Eliminating health disparities should be a goal in relation to CVD mortality reduction in the region.

## CONCLUSION

Mortality rates due to CVD have been decreasing since 2000 in the North American, Latin American and Non-Latin Caribbean regions. Disparities in risk, premature mortality and trends were seen across these three different regions.
